# Supplementary material for: Artificial Diet Assay Screening of Candidate RNAi Effectors Against Myzus persicae (Hemiptera)
Source: Insects. 2025 Oct 23;16(11):1086. doi: 10.3390/insects16111086 (PMC12653897; doi:10.3390/insects16111086)
Supplement: Supplementary file 1 [file insects-16-01086-s001.zip › Supplementary Data S1_V2.pdf]

Supplementary Data 1.

DNA sequences for *in vitro* transcription of dsRNA molecule targeting respective genes in *Myzus persicae*. Highlighted and/or underlined sequences show restriction site *Nco* I (CCATGG) and *Nhe* I (GCTAGC) for bacterial vector cloning

1. Osmoregulatory Aquaporin 1 (AQP) Base pairs 331-830 (500 bp) of GenBank Accession Number: KR047100

Nco I
CCATGGCCAGTGGATGTCATATCAACCCAGCAGTCACGGTTAGCTTTTGGTGAGCGGCCAATGCT
CATTTTTGAAATCTGCTCTGTACATAGTTGCACAGTGTTTGGGGGCTATAGCCGGTGTCTACCTACT
AGAATTTGTTACTCCAGACGCAGTAGCTAAAGGTTTAGGCAAGACAGACGTAAGTCCGCTGCTTC
AACCGGGACAAGGATTCGTCGTAGAAGCATTCATTACCTTTATTCTAATATTGGTTATTCACTCGG
TTTGCGATGAAGCCAGCCGAAGCAACATCGTTACTCCGTCCATCTCCATAGGTCTGACCATTGCTG
CCGCCCATCTAGCAGCGATTAAATACACAGGAGCTAGCATGAATCCAGCAAGATCTTTGGGCCCA
GCTGTTGCTCTTGGTGTATGGTCAAATCATTGGGTGTACTGGGTTGGTCCTATTGTAGGTGGCATAC
TTGGAGGAACCGTCCACACTTTCGTGTTGAAACGTCATACTGAAGCTAGC
Nhe I

2. Osmoregulatory Sucrose transporter (SUC). Base pair 1334 – 1683 (350 bp) of GenBank Accession Number KR047101

CCATGGGACAGTTCGTATAACGCAGGTTTTTCTAATTCATCTTCGTTGTGGCTTCCTCTTAACTCCG
ATTATTGGAAAAAAAATATGGTAGAAGAATCAAGGTATAAAAGTAACCTAAGGTCGTACAGGCAA
TTGGCCCGGTTAAGGAGGAGTCTGACTTTTGTCAAAGGCGATTTGCATCTATACACACTGTCCAAA
TGGGTGTTTGGATTTTCAAGGAGTTTTTATGATCACCCGACTTACTTCATCGTAGTTAACTTTGGTA
GTGAAATAGAAACAGTTAACTTGTTGGAAGCTAGAGGTACTTTACCACTGACTTTGAAAGTTAAG
GTCTCTAGTATTAACTCTGGTTATGTGCTAGC

3. Acetcholinesterase (ACE) Base pairs 1600 – 1999 (400 bp) of GenBank Accession Number: KJ561353

CCATGGCCGAAATGGACAGGGGTGATGCACGGTGATGAAATCAGTTATGTATTTGGAGATCCTTT
AAATCCAAATAAACGCTACGAAATCGAAGAAATCGAACTTAGCAAGAAAATGATGAGATACTGG
ACCAATTTTGCAAAAACAGGAAATCCAAGCAAAACATTGAAGTCTTGGGTTACACGCCAGTGGCC
CGTACACACGGCGTATGGAAAAGAGTTTCTAACATTAGATACAAATAACACTTCTATCGCGTGCA
AGACTAGACAATGTGCTTTTTGGAAAAACTACGTTCCTGATCTTATGGCCATTTCAAAGAGTATGA
AGTCTGACAAAAACTGTACAACCATAAGTGGAGGGACCAAAACTTACATGATTGAGTTATCGCTT
TGGACAATTGTGATGAGCTAGC

4. Salivary glands (C002) Base pairs 391-890 (500 bp) of GenBank Accession Number: EC389531.1

CCATGGTAAGGAATTGGGCTCCAACGAAGTGTGCTCGGACACGACCCGGGCCTTATTTAACTTCGT
CGATGTGATGGCCACCAGCCCGTACGCCCACTTCTCGCTAGGTATGTTTAACAAGATGGTGGCGTT
TATTTTGAGGGAGGTGGACACGACATCGGACAAATTTAAAGAGACGAAGCAGGTGGTCGACCGTA
TCTCGAAAACTCCAGAGATCCGTGACTATATCAGGAACTCGGCCGCCAAGACCGTCGACTTGCTCA
AGGAACCCAAGATTAGAGCACGACTGTTCAGAGTGATGAAAGCCTTCGAGAGTCTGATAAAACCA
AACGAAAACGAAGCATTAATCAAACAGAAGATTAAGGGGTTAACCAATGCTCCCGTCAAGTTAGC
CAAGGGTGCCATGAAAACGGTTGGACGTTTCTTTAGACATTTTTAATAAGCACGTCCATATAGACT
AGTACTATATACTATATATATATATACTTAAAACATAGTACATAAAGGCTAGC

5. Transcription factor Glial (Tfglial) Base pairs 588 - 853 (266 bp) of GenBank Accession Number: XM_022324935

CCTACCCGACGACGCAATAACTATCGGCCCGGCCGCCGCGGAGAGCAGCACGCTCGATCTGAGAC
CGCCGACGAAGGGCAGACCGGACTTCGATTATGGTCGTCATTTTTAAACAAGAATACGAAAATCA
AAGCATGAGCAATGAATATAGTTACTCTCGTGGAAGACCTCCTGCAGAATCTATTTCTCGAATTGC

AGATTGGGACATTAACGATTCGATTATTCCAAGGGTGAGTAACTTCAACTTGTGGGAAGAATGGG
CTGAC

6. Proteasome Subunit α (ProtSubAlpha) Base pairs 260 - 569 (310 bp) of GenBank Accession Number: XM_022311085

TGGAGTTCTCCTTGCTGCTGAAAAACGGAATGTGAATAAACTGCTCGATGAGACTTGTGGATCAGA
AAAAATTTATAAATTAAATGATGACATGGTTTGCAGTGTAGCTGGTATTACTGCTGATGCTAATGTC
TTGGTTAGTGAGTTACGTTCTATTTCAGAACGTTATCGTATGCAGTATGGAGATTCTATACCGTGTG
AACAGCTTGTTTCATGGTTGTGTGATGTAAAACAAGCATATACTCAATATGGAGGTAAAAGACCAT
TTGGTGTATCTATATTATATATGGGTTGGGATTCACATTATGGC

7. S-adenosylmethionine synthase (S-AdMethSynth) Base pairs 622 - 871 (250 bp) of GenBank Accession Number: XM_022314690

TATTGCGGATGGTGTACACGTTAACAAAATTGAAGATGCAATTGGGGCTGGGGATCAGGGCCTCA
TGTTTGGCTATGCTACAGATGAAACAGAAGAATGTATGCCTTTAACAGTGGTACTTGCGCACAAGC
TTAATGAAAAAGTCGCTGAACTCCGAAGAAATGGAGTACTCTGGTGGGCACGTCCTGACACTAAA
ACACAGGTAACATGTGAATACTGTTTGGTGGGCGGTGCTTGCATTCCACAAAGA

8. ATP-dependent RNA helicase (RNAHelicase) Base pairs 667 - 994 (328 bp) of GenBank Accession Number: XM_022316612

AAAGGCTATGTCCGACGAGGAAGTTCAGTCATTCAGAACTGCTCAAAACGAGATTACCGTTAAGT
ATGTGGATGGCGCTGAATATACCCGAACAATACCGAAGCCAGTAAAAACATTTGAGCATGCTTTT
AGCTCATATCCAGATATCATGAAAGTGATTAAAAAACAAAATTTTACCACACCCTCGCCCATTCAG
TGTCAAGCCTGGCCTATAATTATGAGTGGACATGACTTAATAGCCATTGCGCAAACGGGTACAGG
CAAGACATTGGCATACATACTGCCAGCTTTAATTCATTTGATTCAACAGCCCACTCCACGCAATAA
A

9. Concatomer 830 Osmoregulatory Aquaporin 1 (AQP), Osmoregulatory Sucrose transporter (MpSuc1), Acetylcholinesterase (ACE), Salivary glands (C002). GCTAGC

AGCAGCGATTAAATACACAGGAGCTAGCATGAATCCAGCAAGATCTTTGGGCCCAGCTGTTGCTC
TTGGTGTATGGTCAAATCATTGGGTGTACTGGGTTGGTCCTATTGTAGGTGGCATACTTGGAGGAA
CCGTCCACACTTTCGTGTTGAAACGTCATACTGAA**TGTCCAAATGGGTGTTTGGATTTTCAAGG**
AGTTTTTATGATCACCCGACTTACTTCATCGTAGTTAACTTTGGTAGTGAAATAGAAACAGTT
AACTTGTTGGAAGCTAGAGGTACTTTACCACTGACTTTGAAAGTTAAGGTCTCTAGTATTAAC
TCTGGTTATGTTTATTATTATTATTTTTTAAACTATTTATATTGTAAATTTCAATATATCATAAAGA
AATAATTACATAATAGGTATTATACATCATCAGATAAATAATATATTATAATAATGCCCTTTCTAA
TAGATATAATTTAACCGAATGCATTTCTATTCCTGAAATGAATT**AACACTTCTATCGCGTGCAAG**
ACTAGACAATGTGCTTTTTGGAAAAACTACGTTCCTGATCTTATGGCCATTTCAAAGAGTATG
AAGTCTGACAAAAACTGTACAACCATAAGTGGAGGGACCAAAACTTACATGATTGAGTTATC
GCTTTGGACAATTGTGATGAGCATTAATCAAACAGAAGATTAAGGGGTTAACCAATGCTCCCGT
CAAGTTAGCCAAGGGTGCCATGAAAACGGTTGGACGTTTCTTTAGACATTTTTAATAAGCACGTCC
ATATAGACTAGTACTATATACTATATATATATATACTTAAAACATAGTACATAAAGGCTAGC
