# Supplementary material for: Artificial Diet Assay Screening of Candidate RNAi Effectors Against Myzus persicae (Hemiptera)
Source: Insects. 2025 Oct 23;16(11):1086. doi: 10.3390/insects16111086 (PMC12653897; doi:10.3390/insects16111086)
Supplement: Supplementary file 1 [file insects-16-01086-s001.zip › Supplementary Table S1.pdf]

Supplementary Table 1: Summary of reported RNAi studies in *Aphididae* pests. (Modified from [1])

Species	Target Genes	Gene Location and Function	Life Stage of insect	Delivery	dsRNA length (bp)	[Conc] of dsRNA	mRNA Silencing (%) and duration	RNAi duration and effects	Reference
**Pea aphid *Acyrthosiphon pisum***	Gap gene *Hunchback*	Embryo, Role in insect axial patterning	Adult	Injection	448	6-1200 ng	70%, 36 hours	4.5 days, 25% mortality	[2]
	Chitin synthase	Whole body, Growth and development	Nymphs, Adults	Injection and ingestion	364	300-600 ng, 800-1200 ng/ul	50%, 36 hours 35%, 36 hours	3 days, 57% mortality, Molting defects	[3]
	Salivary protein *COO2*	Salivary glands, Role in feeding	Adult	Injection	21-23	50 ng per adult	100%, 4 days	8 days, 100% mortality	[4]
	Enzyme *Cathepsin*-L	Gut, Digestive enzyme	3rd instar	Injection and feeding	357	2-10 µg/ul, 0.9-2.6 µg/µl	Lower expression level, 5 days	5 days, 31% mortality, 5 days, 46% mortality	[5]
	Aquaporin *ApAQP1*	Stomach and intestine, Osmoregulation	Adults	Feeding	451	1 µg/ul	Lower expression level, 4 days	Elevated osmotic pressure of hemolymph	[6]
	Angiotensin-converting enzymes *ACE1, ACE2*	Saliva, Modulates aphid-plant interaction	Third instar	Injection	313, 468	138 ng	73-85%	8 days, 60% mortality	[7]
**Brown citrus aphid *Aphis citridus***	Insulin receptor genes *AcInR1* and *AcIn R2*	Growth and development	Fourth instar	Feeding	511, 609	3000 ng/ul	64-72%, 3 days	62% mortality, Developmental defects	[8]

**Cotton aphid** ***Aphis gossypii***	Carboxylesterase *CarE*	Gut, Role in metabolism of xenobiotic	Third instar	Feeding	686	50-500 ng/ul	33%, 3 days	Decreased resistance to organophosphorus insecticides	[9]
	Odorant – binding proteins *AgOBP2*	Antennae, Role in host-seeking	Third instar	Feeding	434	62.5-250 ng/ul	55-7%, 4 days	Impaired response to physiologically relevant compound	[10]
	Cytochrome P450 monooxygenase gene *CYP6A2*	Gut, Detoxification	Adult, Third instar	Feeding	773	100 ng/ul	Lower expression level, 2-3 days	Increased sensitivity to spirotetramat and alpha-cypermethrin	[11]
**Glasshouse-potato aphid** ***Aulacorthum solani***	*Galanthus nivalis* agglutinin, *GNA*	Plant defensive proteins	Adult	Transgenic potato	-	-	-	Decreased fecundity, Insecticidal activity	[12]
**Mustard aphid** ***Lipaphis erysimi***	*Allium cepa* L. agglutinin, *ACA*	Plant defensive proteins	First instar nymphs	Transgenic Mustard	342	-	-	Decreased fecundity, Insecticidal activity, 14 days	[13]
**Vetch aphid** ***Megoura viciae***	Tyrosine hydroxylase *MV-TH*	Cuticle, Role in tanning	4th instar	Injection	400	1200 ng	Lower expression level, 3 days	Decrease in exuvia tanning, 3 days	[14]
**Tobacco aphid** ***Myzus nicotianae***	*TRV-ALY* and *TRV-Eph*	Whole body, Role in virus transmission	2nd instar	Transgenic *Nicotiana benthamiana*	182, 249	-	19-56%, 7 days<br>12-15%, 7 days	-	[15]
**Green peach aphid** ***Myzus persicae***	Cuticular protein *MyCP*	Cuticle, Molting	3-5 day old nymphs	Transgenic *Arabidopsis thaliana*	327	-	Lower expression level, 14 days	40-47% Decrease in fecundity, 8 and 15 dpi	[16]
	Voltage-gated sodium channels *MpNa$_v$*	Whole body, Transmission of nerve impulses	3rd instar	Feeding	289	750 ng/ul	Lower expression level, 14 days	65% mortality, lower fecundity and longevity, 7 days	[17]

	GPA *Rack1*	Whole body, Development	0-2 day old nymphs	Transgenic *A. thaliana*	309	-	70%, 8 days	30-40% mortality, 4 weeks	[18]
**Bird cherry-oat aphid *Rhopalosiphum padi***	Acetylcholinesterase gene *RpAce1*	CNS, Mediates synaptic transmission	2 day old adult aphid	Injection	383-400	10 ng	63.5%, 2 days	Increased susceptibilities to pirimicarb and malathion, and reduced fecundity	[19]
**Wheat aphid *Schizaphis graminum***	*MRA, GAT, TLP*	Intestine, Role in detoxification	3rd instar	Feeding	376, 433, 422	50 ng/ul	49-55%, 2 days	Increased susceptibility to imidacloprid	[20]
**Grain aphid *Sitobion avenae***	Zinc finger protein *SaZFP*	Gut, Involve in ingestion and digestion	3rd instar	Feeding, Transgenic Wheat	198	-	Lower expression level, 8 days	80% mortality, 8 days 80%, 18 days, decreased fecundity	[21]
	G proteins *Gqα*	Whole body, Involve in olfaction, sight	3rd instar, Nymphs	Feeding, Transgenic Wheat	517	10 ng/ul	Lower expression level, 4 days	Reduction in reproduction and molting, 7 days	[22]
	Unigenes DSR32, DSR33, DSR48	Salivary gland and gut, Role in digestion	3rd instar	Feeding	200-500	10 ng/ul	Lower expression level, 8 days	60-100% mortality, 8 days	[7]
	Catalase CAT	Midgut, Scavenging reactive oxygen	3rd instar	Feeding	471	3-50 ng/ul	40%, 6 days	60% mortality, 6 days	[23]

References

1. Jain, R.G.; Robinson, K.E.; Asgari, S.; Mitter, N. Current scenario of RNAi-based hemipteran control. *Pest Manag Sci* **2021**, *77*, 2188-2196, doi:https://doi.org/10.1002/ps.6153.
2. Ye, C.; An, X.; Jiang, Y.-D.; Ding, B.-Y.; Shang, F.; Christiaens, O.; Taning, C.N.T.; Smagghe, G.; Niu, J.; Wang, J.-J. Induction of RNAi core machinery's gene expression by exogenous dsRNA and the effects of pre-exposure to dsRNA on the gene silencing efficiency in the Pea aphid (*Acyrthosiphon pisum*). *Frontiers in Physiology* **2019a**, *9*, doi:https://doi.org/10.3389/fphys.2018.01906.
3. Ye, C.; Jiang, Y.-D.; An, X.; Yang, L.; Shang, F.; Niu, J.; Wang, J.-J. Effects of RNAi-based silencing of chitin synthase gene on moulting and fecundity in pea aphids (*Acyrthosiphon pisum*). *Scientific Reports* **2019b**, *9*, 3694, doi:https://doi.org/10.1038/s41598-019-39837-4.
4. Mutti, N.S.; Park, Y.; Reese, J.C.; Reeck, G.R. RNAi knockdown of a salivary transcript leading to lethality in the pea aphid, *Acyrthosiphon pisum*. *J Insect Sci* **2006**, *6*, 1-7, doi: https://doi.org/10.1673/031.006.3801.
5. Sapountzis, P.; Duport, G.; Balmand, S.; Gaget, K.; Jaubert-Possamai, S.; Febvay, G.; Charles, H.; Rahbé, Y.; Colella, S.; Calevro, F. New insight into the RNA interference response against cathepsin-L gene in the pea aphid, *Acyrthosiphon pisum*: molting or gut phenotypes specifically induced by injection or feeding treatments. *Insect Biochemisrtry and Molecular Biology* **2014**, *51*, 20-32, doi: https://doi.org/10.1016/j.ibmb.2014.05.005.
6. Shakesby, A.J.; Wallace, I.S.; Isaacs, H.V.; Pritchard, J.; Roberts, D.M.; Douglas, A.E. A water-specific aquaporin involved in aphid osmoregulation. *Insect Biochem Mol Biol* **2009**, *39*, 1-10, doi:https://doi.org/10.1016/j.ibmb.2008.08.008.
7. Wang, W.; Luo, L.; Lu, H.; Chen, S.; Kang, L.; Cui, F. Angiotensin-converting enzymes modulate aphid–plant interactions. *Scientific Reports* **2015**, *5*, 8885, doi:https://doi.org/10.1038/srep08885.
8. Ding, B.Y.; Shang, F.; Zhang, Q.; Xiong, Y.; Yang, Q.; Niu, J.Z.; Smagghe, G.; Wang, J.J. Silencing of two insulin receptor genes disrupts nymph-adult transition of alate brown citrus aphid. *International Journal of Molecular Sciences* **2017**, *18*, 357, doi:https://doi.org/10.3390/ijms18020357.
9. Gong, Y.H.; Yu, X.R.; Shang, Q.L.; Shi, X.Y.; Gao, X.W. Oral delivery mediated RNA interference of a carboxylesterase gene results in reduced resistance to organophosphorus insecticides in the cotton Aphid, *Aphis gossypii* Glover. *PLoS One* **2014**, *9*, e102823, doi: https://doi.org/10.1371/journal.pone.0102823.
10. Rebijith, K.B.; Asokan, R.; Hande, H.R.; Kumar, N.K.; Krishna, V.; Vinutha, J.; Bakthavatsalam, N. RNA interference of odorant-binding protein 2 (OBP2) of the Cotton Aphid, *Aphis gossypii* (Glover), resulted in altered electrophysiological responses. *Applied Biochemistry and Biotechnology* **2016**, *178*, 251-266, doi:https://doi.org/10.1007/s12010-015-1869-7.
11. Peng, T.; Pan, Y.; Yang, C.; Gao, X.; Xi, J.; Wu, Y.; Huang, X.; Zhu, E.; Xin, X.; Zhan, C.; et al. Over-expression of CYP6A2 is associated with spirotetramat resistance and cross-resistance in the resistant strain of *Aphis gossypii.* Glover. *Pesticide Biochemistry Physiology* **2016**, *126*, 64-69, doi: https://doi.org/10.1016/j.pestbp.2015.07.008.

12. Down, R.E.; Gatehouse, A.M.R.; Hamilton, W.D.O.; Gatehouse, J.A. Snowdrop lectin inhibits development and decreases fecundity of the Glasshouse Potato Aphid (*Aulacorthum solani*) when administered *in vitro* and via transgenic plants both in laboratory and glasshouse trials. *Journal of Insect Physiology* **1996**, *42*, 1035-1045, doi:https://doi.org/10.1016/S0022-1910(96)00065-0.
13. Hossain, M.A.; Maiti, M.K.; Basu, A.; Sen, S.; Ghosh, A.K.; Sen, S.K. Transgenic expression of onion leaf lectin gene in Indian mustard offers protection against aphid colonization. *Crop Science* **2006**, *46*, 2022-2032, doi:https://doi.org/10.2135/cropsci2005.11.0418.
14. Wang, X.X.; Feng, Z.J.; Chen, Z.S.; Zhang, Z.F.; Zhang, Y.; Liu, T.X. Use of tyrosine hydroxylase RNAi to study *Megoura viciae* (Hemiptera: Aphididae) sequestration of its host's l-DOPA for body melanism. *Journal of Insect Physiology* **2019**, *114*, 136-144, doi:https://doi.org/10.1016/j.jinsphys.2019.03.007.
15. Mulot, M.; Boissinot, S.; Monsion, B.; Rastegar, M.; Clavijo, G.; Halter, D.; Bochet, N.; Erdinger, M.; Brault, V. Comparative analysis of RNAi-based methods to down-regulate expression of two genes expressed at different levels in *Myzus persicae*. *Viruses* **2016**, *8*, 316, doi:https://doi.org/10.3390/v8110316.
16. Bhatia, V.; Bhattacharya, R. Host-mediated RNA interference targeting a cuticular protein gene impaired fecundity in the green peach aphid *Myzus persicae*. *Pest Management Science* **2018**, *74*, 2059-2068, doi:https://doi.org/10.1002/ps.4900.
17. Tariq, K.; Ali, A.; Davies, T.G.E.; Naz, E.; Naz, L.; Sohail, S.; Hou, M.; Ullah, F. RNA interference-mediated knockdown of voltage-gated sodium channel (MpNav) gene causes mortality in peach-potato aphid, *Myzus persicae*. *Scientific Reports* **2019**, *9*, 5291, doi: https://doi.org/10.1038/s41598-019-41832-8.
18. Coleman, A.D.; Wouters, R.H.M.; Mugford, S.T.; Hogenhout, S.A. Persistence and transgenerational effect of plant-mediated RNAi in aphids. *Journal of Experimental Botany* **2015**, *66*, 541-548, doi:https://doi.org/10.1093/jxb/eru450.
19. Xiao, D.; Lu, Y.H.; Shang, Q.L.; Song, D.L.; Gao, X.W. Gene silencing of two acetylcholinesterases reveals their cholinergic and non-cholinergic functions in Rhopalosiphum padi and Sitobion avenae. *Pest Management Science* **2015**, *71*, 523-530, doi:https://doi.org/10.1002/ps.3800.
20. Zhang, B.Z.; Ma, K.S.; Liu, J.J.; Lu, L.Y.; Chen, X.L.; Zhang, S.P.; Gao, X.W. Differential expression of genes in greenbug (Schizaphis graminum Rondani) treated by imidacloprid and RNA interference. *Pest Management Science* **2019**, *75*, 1726-1733, doi:https://doi.org/10.1002/ps.5293.
21. Sun, Y.; Sparks, C.; Jones, H.; Riley, M.; Francis, F.; Du, W.; Xia, L. Silencing an essential gene involved in infestation and digestion in grain aphid through plant-mediated RNA interference generates aphid-resistant wheat plants. *Plant Biotechnology Journal* **2019**, *17*, 852-854, doi: https://doi.org/10.1111/pbi.13067.
22. Hou, Q.; Xu, L.; Liu, G.; Pang, X.; Wang, X.; Zhang, Y.; You, M.; Ni, Z.; Zhao, Z.; Liang, R. Plant-mediated gene silencing of an essential olfactory-related Gqα gene enhances resistance to grain aphid in common wheat in greenhouse and field. *Pest Management Science* **2019**, *75*, 1718-1725, doi: https://doi.org/10.1002/ps.5292.
23. Deng, F.; Zhao, Z. Influence of catalase gene silencing on the survivability of *Sitobion avenae*. *Archives of Insect Biochemistry and Physiology* **2014**, *86*, 46-57, doi:https://doi.org/10.1002/arch.21161.
