# Supplementary material for: Artificial Diet Assay Screening of Candidate RNAi Effectors Against Myzus persicae (Hemiptera)
Source: Insects. 2025 Oct 23;16(11):1086. doi: 10.3390/insects16111086 (PMC12653897; doi:10.3390/insects16111086)
Supplement: Supplementary file 1 [file insects-16-01086-s001.zip › Supplementary Table S2_V4.pdf]

Supplementary Table 2: Primers used in this study

Name	Sequence 5' – 3' †	Tm °C	Length
**In vitro dsRNA Synthesis Primers**			
MpUniT7-fwd	GAATTC**TAATACGACTCACTATAGGG**CCAT<br>*Eco*RI	57.8	500bp
MpAQPT7-rev	GATCG**TAATACGACTCACTATAGGG**CTTCAGTATGACGTTTC<br>*Nhe*I	64.9	350bp
MpSUCT7-rev	GATCG**TAATACGACTCACTATAGGG**ACATAACCAGAGTTAATA	62.5	500bp
MpACET7-rev	GATCG**TAATACGACTCACTATAGGG**TCATCACAATTGTCCAAAG	64.9	500bp
MpC002&830T7-rev	GATCG**TAATACGACTCACTATAGGG**CTTTATGTACTATGTTTTA	61.9	830bp
Tfglial-fwd	**TAATACGACTCACTATAGGG**CCTACCCGACGACGCAATAA	67	266bp
Tfglial-rev	**TAATACGACTCACTATAGGG**GTCAGCCCATTCTTCCCACA	68	
S-AdMethSynth-fwd	**TAATACGACTCACTATAGGG**TATTGCGGATGGTGTACACGT	66	250bp
S-AdMethSynth-rev	**TAATACGACTCACTATAGGG**TCTTTGTGGAATGCAAGCACC	66	
ProtSubAlpha-fwd	**TAATACGACTCACTATAGGG**TGGAGTTCTCCTTGCTGCTG	67	310bp
ProtSubAlpha-rev	**TAATACGACTCACTATAGGG**GCCATAATGTGAATCCCAACCC	66	
RNAHelicase-fwd	**TAATACGACTCACTATAGGG**AAAGGCTATGTCCGACGAGG	67	328bp
RNAHelicase-rev	**TAATACGACTCACTATAGGG**TTTATTGCGTGGAGTGGGCT	67	

RT-qPCR primers

Name	Sequence 5' – 3' †	Efficiency	Tm °C	Length
rtMpAQP-F	TGGAGAATGCTGTTTGCCGA	106.3%	67	229bp
rtMpAQP-R	AAAATGAGCATTGGCCGCTC		67	
rtMpSUC-F	TTAATGTTGGCGCCGTTTGG	93.84%	67	280bp
rtMpSUC-R	CCACGTGAATCTATGACCGGA		66	
rtMpACE-F	TCCATGGCCGAAATGGACAG	99.57%	68	264bp
rtMpACE-R	GTCTTGCACGCGATAGAAGTG		66	
rtMpC002-F	TTTTGAGGGAGGTGGACACG	98.59%	67	278bp
rtMpC002-R	CGTTTTCATGGCACCCTTGG		67	
qRNAHelicase_F	TGAGTGCAGATGCATACATGG	101.31%	65	83bp
qRNAHelicase_R	TGGATACAACACCATTGCGTAT		64	
qTfglial_F	GTCCACCTAAGACCAGCTATATG	108.69%	64	113bp
qTfglial_R	CAGTGTCCTCTACAAGGCATTA		64	
qProtSubAlpha_F	CTGCTGCCATTGCTTCATT	92.13%	64	143bp
qProtSubAlpha_R	AAGTGTAGCCATTTCCACTTTATC		62	
qS-AdMethSynth_F	TCGTCTCAGTTGATGAACAGTC	97.52%	64	100bp
qS-AdMethSynth_R	TCATCTGTAGCATAGCCAAACA		63	
Rpl7-F	TGCCGGAGTCTGTACTCAAA	96.93%	66	92bp
Rpl7-R	CACGCGTTCTTTACGTTCCT		65	
18S-F	GATCGTGGCTTGCAATTTTT	93.07%	60.1	91bp
18S-R	GTACAAAGGGCAGGGACGTA		60.0	

† Bold sequences represent T7 polymerase recognition site. Restriction endonuclease sites *Eco*RI (GAATTC) and *Nhe*I (GATCG) facilitate vector cloning
